# Use of Leukotriene-Receptor Antagonists During Pregnancy and Risk of Neuropsychiatric Events in Offspring

**DOI:** 10.1001/jamanetworkopen.2023.1934

**Published:** 2023-03-07

**Authors:** Hui-Ju Tsai, Chi-Shin Wu, Yen-Chen Chang, Tsung-Chieh Yao

**Affiliations:** 1 Institute of Population Health Sciences, National Health Research Institutes, Zhunan, Taiwan; 2 Department of Medical Science, National Tsing Hua University, Hsinchu, Taiwan; 3 National Center for Geriatrics and Welfare Research, National Health Research Institutes, Zhunan, Taiwan; 4 Department of Psychiatry, National Taiwan University Hospital, Yunlin Branch, Douliu, Taiwan; 5 Division of Allergy, Asthma, and Rheumatology, Department of Pediatrics, Chang Gung Memorial Hospital, Taoyuan, Taiwan; 6 School of Medicine, Chang Gung University College of Medicine, Taoyuan, Taiwan

## Abstract

This cohort study examines the association of the use of leukotriene-receptor antagonists during pregnancy with the risk of neuropsychiatric events in offspring.

## Introduction

Leukotriene-receptor antagonists (LTRAs) are a class of medications used for treating allergic airway diseases, including asthma and allergic rhinitis.^1^ Over the years, the US Food and Drug Administration has monitored postmarketing data about the potential harm of neuropsychiatric events (NEs) associated with montelukast, the first type of LTRAs, and issued boxed warnings about serious NEs associated with montelukast use in 2020.^2^ However, evidence regarding the risk of NEs associated with LTRAs has been conflicting.^3,4,5^ To the best of our knowledge, no previous studies have reported the association of exposure to LTRAs during pregnancy with the risk of NEs in offspring. The present cohort study aimed to address this research question.

## Methods

We used data from the entire National Health Insurance Research Database to identify pregnant women and their offspring from 2009 to 2019 in Taiwan. The details of study cohort identification are provided in the eAppendix in Supplement 1. The study protocol was approved by the institutional review board of the National Health Research Institutes, Taiwan, which waived the need for informed consent because the data are anonymous. This study follows the Strengthening the Reporting of Observational Studies in Epidemiology (STROBE) reporting guideline for cohort studies.

The inclusion criteria were pregnant women with a diagnosis of asthma or allergic rhinitis, without multiple births, and their offspring without congenital malformations (eAppendix in Supplement 1). Exposure was defined as having any dispensed prescription for LTRAs during pregnancy. Propensity score matching was applied to control for the systematic differences at baseline between LTRA users and nonusers (eAppendix in Supplement 1). The main outcomes are primary diagnoses of attention-deficit/hyperactivity disorder (ADHD), autism spectrum disorder (ASD), or Tourette syndrome in offspring. We identified the outcomes using *International Classification of Diseases, Ninth Revision, Clinical Modification* codes for 2009 to 2015 and *International Statistical Classification of Diseases, Tenth Revision, Clinical Modification* codes for 2016 to 2019 (eTable in Supplement 1).

Cox proportional hazards models were constructed to estimate the associations between prenatal LTRA exposure and NEs among offspring with covariate adjustment. A robust sandwich estimator was used to account for the dependences between siblings. We also performed Cox proportional hazards models to investigate the association between the duration of LTRA use (1-4 weeks vs >4 weeks) and cumulative LTRA dose (1-170 mg vs >170 mg) and NEs among offspring. The adjusted covariates are described in the eAppendix in Supplement 1. *P* < .05 was considered to denote statistical significance. Data analyses were performed from January to September 2022 using SAS statistical software version 9.4 (SAS Institute).

## Results

A total of 576 157 mother-offspring pairs (1995 LTRA-exposed children and 574 162 nonexposed children) were identified in the original study population. After propensity score matching, 1988 LTRA-exposed children and 19 863 nonexposed children were included in the subsequent analyses (Table). Among the offspring, no significant associations were found between prenatal LTRA exposure and ADHD (adjusted hazard ratio [AHR], 1.03; 95% CI, 0.79-1.35), ASD (AHR, 1.01; 95% CI, 0.65-1.59), and Tourette syndrome (AHR, 0.63; 95% CI, 0.29-1.36) (Figure). Duration of LTRA use (1-4 weeks vs >4 weeks) and cumulative dose of LTRA (1-170 mg vs >170 mg) were not significantly associated with ADHD, ASD, and Tourette syndrome among offspring (Figure).

**Table.** Baseline Demographic and Clinical Characteristics of Women With and Without Use of LTRAs in Pregnancy

Characteristic	Participants, No. (%) (N = 576 157)		Standardized mean difference	
	LTRA use (n = 1995)	No LTRA use (n = 574 162)	Before propensity score matching	After propensity score matching
Age at delivery, y				
<30	555 (27.82)	189 094 (32.93)	0.1114	0.0133
30-40	1315 (65.91)	359 041 (62.53)	0.0706	0.0012
>40	121 (6.07)	22 569 (3.93)	0.0981	0.0222
Unemployment	552 (27.67)	139 787 (24.35)	0.0758	0.0142
Urbanization				
Low	137 (6.87)	30 998 (5.40)	0.0612	0.0062
Median	552 (27.67)	165 771 (28.87)	0.0267	0.0050
High	1300 (65.16)	373 486 (65.05)	0.0024	0.0014
Insured amount, NTD				
<20 000	323 (16.19)	98 681 (17.19)	0.0267	0.0047
20 000 to <40 000	1184 (59.35)	331 978 (57.82)	0.0310	0.0101
40 000 to <60 000	347 (17.39)	102 818 (17.91)	0.0135	0.0118
≥60 000	138 (6.92)	37 407 (6.52)	0.0161	0.0048
Comorbidity				
Cerebrovascular disease	39 (1.95)	6636 (1.16)	0.0646	0.0024
Rheumatic disease	54 (2.71)	13 370 (2.33)	0.0241	0.0014
Peptic ulcer disease	443 (22.21)	99 912 (17.40)	0.1208	0.0068
Mild liver disease	158 (7.92)	34 915 (6.08)	0.0721	0.0182
Diabetes without chronic complication	97 (4.86)	19 615 (3.42)	0.0726	0.0142
Diabetes with chronic complication	21 (1.05)	2389 (0.42)	0.0746	0.0074
Hemiplegia or paraplegia	7 (0.35)	674 (0.12)	0.0483	0.0144
Psychiatric disorders				
Major depressive disorder	82 (4.11)	14 274 (2.49)	0.0910	0.0148
Persistent depressive disorder	148 (7.42)	25 603 (4.46)	0.1255	0.0100
Anxiety disorder	99 (4.96)	19 525 (3.40)	0.0781	0.0061
Panic disorder	38 (1.90)	7247 (1.26)	0.0515	0.0240
Schizophrenia	11 (0.55)	1656 (0.29)	0.0407	0.0034
Bipolar disorder	34 (1.70)	6168 (1.07)	0.0538	0.0192
Cyclothymia	83 (4.16)	13 627 (2.37)	0.1007	0.0031
Substance use disorder	68 (3.41)	11 339 (1.97)	0.0887	0.0080
Adjustment disorder	62 (3.11)	12 721 (2.22)	0.0555	0.0119
Neuropsychiatric events in children				
Attention-deficit/hyperactivity disorder	66 (1.25)	25050 (1.32)	NA	NA
Autism spectrum disorder	25 (3.31)	7567 (4.36)	NA	NA
Tourette syndrome	9 (0.45)	4746 (0.83)	NA	NA

Abbreviations: LTRA, leukotriene-receptor antagonist; NA, not applicable; NTD, new Taiwan dollar.

**Figure.**
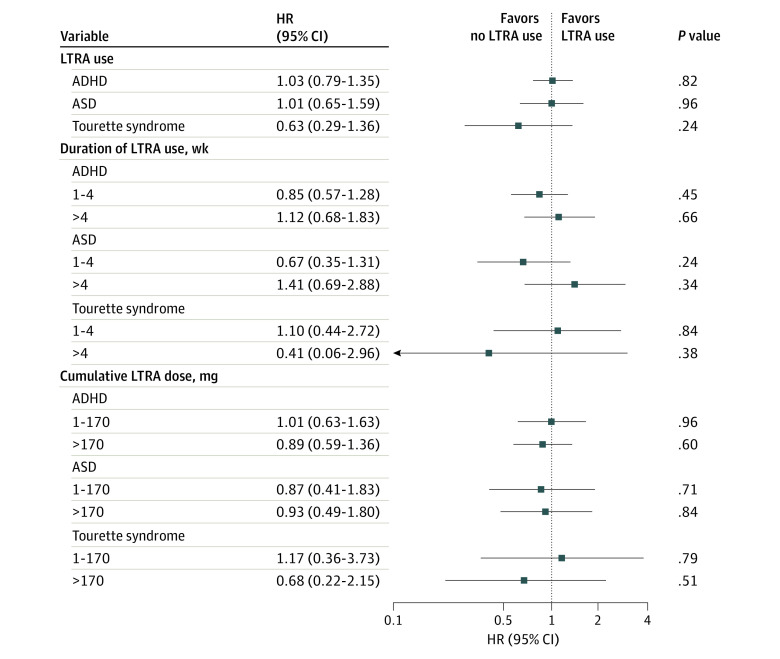
Association of Leukotriene-Receptor Antagonist (LTRA) Use in Pregnancy (Overall, Duration, and Cumulative Dose) With 3 Neuropsychiatric Events in Offspring ADHD indicates attention-deficit/hyperactivity disorder; ASD, autism spectrum disorder; HR, hazard ratio.

## Discussion

This first nationwide cohort study extended previous studies by examining the association between LTRA use during pregnancy and risk of NEs in offspring and found that prenatal LTRA exposure was not associated with increased risk of ADHD, ASD, and Tourette syndrome in offspring. The main strength of this study is the inclusion of the whole Taiwan population data. Study limitations include the nonrandomized design, inability to detect risk beyond the first several years of life, and uncertain generalizability to non-Asian populations.

In conclusion, this study found that LTRA use during pregnancy was not associated with significant risk of NEs in offspring. Clinicians prescribing LTRAs to pregnant women with asthma or allergic rhinitis may be reassured by our findings of no increased risk of NEs in offspring. Our findings should be confirmed by further replication studies.

## Notes

Supplement 1. **eAppendix.** Supplemental Methods
eReferences
**eTable.** *ICD-9-CM* and *ICD-10-CM* Codes of Three Neuropsychiatric Events

Supplement 2.
Data Sharing Statement
