# Supplementary material for: Use of Leukotriene-Receptor Antagonists During Pregnancy and Risk of Neuropsychiatric Events in Offspring
Source: JAMA Netw Open. 2023 Mar 7;6(3):e231934. doi: 10.1001/jamanetworkopen.2023.1934 (PMC9993182; doi:10.1001/jamanetworkopen.2023.1934)
Supplement: Supplement 1. — eAppendix. Supplemental Methods eReferences eTable. ICD-9-CM and ICD-10-CM Codes of Three Neuropsychiatric Events [file jamanetwopen-e231934-s001.pdf]

Supplemental Online Content

Tsai HJ, Wu CS, Chang YC, Yao TC. Use of leukotriene-receptor antagonists during pregnancy and risk of neuropsychiatric events in offspring. *JAMA Netw Open*. 2023;6(3):e231934. doi:10.1001/jamanetworkopen.2023.1934

eAppendix. Supplemental Methods
eReferences
eTable. *ICD-9-CM* and *ICD-10-CM* Codes of Three Neuropsychiatric Events

This supplemental material has been provided by the authors to give readers additional information about their work.

© 2023 Tsai HJ et al. *JAMA Network Open.*

eAppendix. Supplemental Methods

Study Cohort

The study cohort was defined as children born during 2009-2019, ensuring that all children conceived on a given day end up in the cohort,[1] and linked children to their maternal medical claims data using the Maternal and Child Health Database (MCHD). We only included pregnant women with diagnosis of asthma or allergic rhinitis (*ICD-9-CM* codes: 493 and 477 or *ICD-10* codes: J45 and J30 for asthma and allergic rhinitis, respectively) and without multiple births, and their offspring without congenital malformations. Children with congenital malformations were excluded from the subsequent analyses based on the following concerns. First, there is no epidemiologic evidence indicating a causal relationship between leukotriene-receptor antagonists and congenital malformations.[2,3] Secondly, previous studies have documented that children with congenital malformations were associated with risk of neuropsychiatric events (NEs), specifically, attention-deficit/hyperactivity disorder (ADHD), autism spectrum disorder (ASD), and Tourette syndrome.[4-6] In this study, we have aimed to examine the effect of prenatal exposure to leukotriene-receptor antagnoists (LTRAs) on NEs in general pediatric populations. As such, children with congenital malformations were excluded from the subsequent analyses.

We exactly matched by birth year for study children between two groups. Next, ten age-matched individuals in the control group were matched and selected for each LTRA user (at 10: 1 ratio) using propensity scores, which were provided in the following subsection. As

© 2023 Tsai HJ et al. *JAMA Network Open.*

a result, a total of 1,988 LRTA exposed children (age: 4.25 ± 2.94 years) and 19,863 non-exposed children (age: 4.28 ± 2.94 years) were identified for subsequent analyses.

Study Cohort Matched by Propensity Score

To control for the potential confounding effects, we applied a propensity-score method in this study.[7,8] We computed the propensity score based on the predicted probability of each individual taking LTRA conditional on her baseline demographic and clinical conditions and identified a non-user group matched with the LTRA user group with a similar demographic and clinical conditions. Particularly, in a logistic regression model, use of LTRA (yes/no) was regressed on potential confounding factors, such as maternal age at delivery, income, employment status, residential place, maternal comorbidities including cerebrovascular disease, rheumatic disease, peptic ulcer disease, mild liver disease, diabetes with and without chronic complication, hemiplegia or paraplegia, and psychiatric disorders including major depressive disorder, persistent depressive disorder, anxiety disorder, panic disorder, schizophrenia, bipolar disorder, cyclothymia, substance use disorder, and adjustment disorder. Individuals in the control group were adaptively 10: 1 ratio matched by propensity score for each LTRA user. The matching algorithm was without replacement. After propensity score matching, 1,988 LTRA users and 19,863 matched controls were included in the subsequent analyses.

Statistical Analysis

We have defined maternal asthma severity based on maternal subject with previous hospitalization for asthma and previous oral corticosteroids prescription,[9] which could be regarded as a surrogate for asthma severity and were treated as covariates in the models.

Cox proportional hazards models were constructed to estimate the associations between prenatal LTRA exposure and NEs among offspring with covariate adjustment. A robust sandwich estimator was used for accounting the dependences between siblings. We also performed Cox proportional hazards models to investigate the relationship between the effects of duration of LTRA use (1-4 weeks versus >4 weeks), and cumulative drug dose of LTRA (1-170 mg versus >170 mg), and NEs among offspring. The adjusted covariates included previous hospitalization for asthma, previous oral corticosteroids prescription, previous medication treatment for asthma and allergic rhinitis, and co-prescribed medications for asthma and allergic rhinitis. We have assessed the proportional hazards assumptions by including product terms between the predictors and function of survival time in the models. We did not find significant product terms in the models, indicating the assumption was held.

© 2023 Tsai HJ et al. *JAMA Network Open.*

eReferences

1. Strand LB, Barnett AG and Tong S. Methodological challenges when estimating the effects of season and seasonal exposures on birth outcomes. BMC Med Res Methodol. 2011 Apr 18;11:49. doi: 10.1186/1471-2288-11-49.
2. Nelsen LM, Shields KE, Cunningham ML, et al. Congenital malformations among infants born to women receiving montelukast, inhaled corticosteroids, and other asthma medications. J Allergy Clin Immunol. Jan 2012;129(1):251-4 e1-6. doi:10.1016/j.jaci.2011.09.003.
3. Cavero-Carbonell C, Vinkel-Hansen A, Rabanque-Hernández MJ, Martos C, Garne E. Fetal Exposure to Montelukast and Congenital Anomalies: A Population Based Study in Denmark. Birth Defects Research. 2017;109(6):452-459. doi:https://doi.org/10.1002/bdra.23621.
4. Zinkstok JR, Boot E, Bassett AS, Hiroi N, Butcher NJ, Vingerhoets C, Vorstman JAS, van Amelsvoort TAMJ. Neurobiological perspective of 22q11.2 deletion syndrome. Lancet Psychiatry. 2019 Nov;6(11):951-960. doi: 10.1016/S2215-0366(19)30076-8.
5. Gonzalez VJ, Kimbro RT, Cutitta KE, Shabosky JC, Bilal MF, Penny DJ, Lopez KN. Mental Health Disorders in Children With Congenital Heart Disease. Pediatrics. 2021 Feb;147(2):e20201693. doi: 10.1542/peds.2020-1693.
6. Hartshorne TS, Heussler HS, Dailor AN, Williams GL, Papadopoulos D, Brandt KK.

© 2023 Tsai HJ et al. *JAMA Network Open.*

Sleep disturbances in CHARGE syndrome: types and relationships with behavior and caregiver well-being. Dev Med Child Neurol. 2009 Feb;51(2):143-50. doi: 10.1111/j.1469-8749.2008.03146.x.

7. Glynn RJ, Schneeweiss S, Sturmer T. Indications for propensity scores and review of their use in pharmacoepidemiology. Basic Clin Pharmacol Toxicol. 2006 Mar;98(3):253-9.

8. Austin PC. An Introduction to Propensity Score Methods for Reducing the Effects of Confounding in Observational Studies. Multivariate Behav Res. 2011 May;46(3):399-424.

9. Shi T, Pan J, Katikireddi SV, McCowan C, Kerr S, Agrawal U, Shah SA, Simpson CR, Ritchie LD, Robertson C, Sheikh A, on behalf of Public Health Scotland and the EAVE II Collaborators . Risk of COVID-19 hospital admission among children aged 5–17 years with asthma in Scotland: a national incident cohort study. Lancet Respir Med. 2022 Feb;10(2):191-198. doi: 10.1016/S2213-2600(21)00491-4.

© 2023 Tsai HJ et al. *JAMA Network Open.*

eTable. *ICD-9-CM* and *ICD-10-CM* Codes of Three Neuropsychiatric Events

**Diagnosis**	***ICD-9-CM* Code**	***ICD-10-CM* Code**
Attention-deficit/hyperactivity disorder	314.xx	F90
Autism spectrum disorder	299.xx	F84
Tourette syndrome	307.2x	F95

Abbreviation: ***ICD-9-CM****: International Classification of Diseases, Ninth Revision, Clinical Modification*; ***ICD-10-CM****: International Classification of Diseases, Tenth Revision, Clinical Modification*.

© 2023 Tsai HJ et al. *JAMA Network Open.*
