# Supplementary material for: Use of Leukotriene-Receptor Antagonists During Pregnancy and Risk of Neuropsychiatric Events in Offspring
Source: JAMA Netw Open. 2023 Mar 7;6(3):e231934. doi: 10.1001/jamanetworkopen.2023.1934 (PMC9993182; doi:10.1001/jamanetworkopen.2023.1934)
Supplement: Supplement 2. — Data Sharing Statement [file jamanetwopen-e231934-s002.pdf]

Data Sharing Statement

Tsai. Use of Leukotriene-Receptor Antagonists During Pregnancy and Risk of Neuropsychiatric Events in Offspring. *JAMA Netw Open*. Published March 07, 2023.
doi:10.1001/jamanetworkopen.2023.1934

Data
Data available: No
